# The influence of STEM definitions for research on women’s college attainment

**DOI:** 10.1186/s40594-018-0144-1

**Published:** 2018-11-01

**Authors:** Catherine A. Manly, Ryan S. Wells, Suzan Kommers

**Affiliations:** 1 Department of Educational Policy, Research and Administration, University of Massachusetts Amherst, N119 Furcolo Hall, 813 North Pleasant Street, Amherst, MA 01003 USA; 2 Department of Educational Policy, Research and Administration, University of Massachusetts Amherst, N172 Furcolo Hall, 813 North Pleasant Street, Amherst, MA 01003 USA

**Keywords:** STEM definition, Women, Gender, College attainment

## Abstract

**Background:**

Prior research has inconsistently operationalized Science, Technology, Engineering, and Math (STEM) fields, presenting an interpretation challenge. A content analysis of 51 quantitative, gender-focused, higher education-oriented, STEM-related studies in the ERIC database published between January 2010 and July 2018 revealed that only 13 articles used an existing STEM definition. In 15, STEM was not explicitly defined, and others defined STEM independently. This wide range of definitions may lead to confusion or misrepresentation of findings for interventions and practices to support women in STEM. To illustrate the issue and prompt recommendations for future research, this study uses data from the United States National Center for Education Statistics’ Education Longitudinal Study (ELS:2002/12) to investigate the connection between STEM definition and the outcome of college degree completion, comparing results by gender for five ways of operationalizing STEM fields.

**Results:**

We found the size, direction, and significance of the gender gap depended on STEM operationalization. When STEM was defined as high paradigm fields, the odds of women attaining a non-STEM degree were higher than otherwise. When social science fields were included in STEM, there was no statistically significant difference by gender. When looking specifically at fields considered related to science and engineering, the direction of the relationship was reversed.

**Conclusion:**

While our findings follow expectations about social science fields and gender, it is noteworthy that results regarding STEM degree completion by gender for science and engineering-related fields were opposite those of high paradigm STEM fields. This result highlights that the definition of STEM matters, and inconsistent operationalization in the literature presents an interpretation challenge. We argue the field should strive to find common categorizations of STEM that retain the legitimate variation in how STEM can and should be defined, while providing a basis for consistent comparison. We recommend researchers and practitioners developing research-based practices: 1) interpret research findings understanding potential inconsistency from different STEM operationalizations, 2) explicitly describe STEM operational definitions to enable comparing findings, 3) routinely analyze sensitivity to alternate STEM definitions, and 4) find common STEM categorizations that retain legitimate variation while providing a basis for consistent comparison.

**Electronic supplementary material:**

The online version of this article (10.1186/s40594-018-0144-1) contains supplementary material, which is available to authorized users.

Given wide recognition of the importance of science, technology, engineering, and mathematics (STEM) education and the need to support students through STEM degree pathways that will fill national workforce needs (National Science Board 2015), equity concerns for underrepresented groups, such as people of color, individuals with disabilities, and particularly women, represent an issue of ongoing importance (National Science Foundation, National Center for Science and Engineering Statistics 2015). Diminished STEM access or degree completion for these groups limits opportunities for well-paying, high-status jobs, likely maintaining or exacerbating social inequality, particularly given restrictive access, demanding expectations, and opportunities for such jobs upon degree completion.

Past studies with a focus on women in STEM have examined women’s STEM-major choice (Davison et al. 2014), predictors of a STEM major such as academic preparation and/or STEM attitudes (Riegle-Crumb and King 2010), and the climate and sense of belonging of women in STEM (Johnson 2012; Rincón and George-Jackson 2016), among many others. However, to be able to understand the effectiveness of efforts to improve STEM outcomes for women, scholars must first reliably be able to understand what is meant by STEM. Scholars have noted that the designation of STEM areas is an evolving issue (Ackerman et al. 2013) which has not yet led to an agreed-upon classification of STEM fields (Zhang 2011), but the issue is not often articulated or understood. To make valid claims about ways institutions of higher education can support women in their studies throughout their trajectory to STEM degree completion and beyond (e.g., Gayles and Ampaw 2014), consistent and transparent definitions of STEM are critical in research on college students. Unfortunately, however, these characteristics are elusive in existing quantitative STEM education research, including research on gender and STEM.

This inconsistency in STEM operational definitions was revealed through a content analysis of peer-reviewed journal articles in the ERIC education database. A review of 51 quantitative, gender-focused, higher education-oriented, STEM-related studies published between January 2010 and July 2018 (see Additional file 1 for additional details) revealed that in 13 instances, authors used an existing definition for STEM such as that from the National Science Foundation (NSF) or UNESCO’s International Standard Classification of Education. In 23 studies, authors operationally defined STEM, but without an external reference. In 15 of the articles, STEM was not explicitly defined at all. Even when leveraging external definitions, however, there are distinct ways of defining STEM that must be made clear for comparability across studies. For instance, one definition used by the National Center for Education Statistics (NCES; Chen and Weko 2009) strictly includes “mathematics; natural sciences (including physical sciences and biological/agricultural sciences); engineering/engineering technologies; and computer/information sciences” (p. 2), while the definition from the NSF more broadly defines STEM by including social and behavioral sciences.

Given the prevalence of inconsistent and/or unreported STEM definitions, we posit that literature on gender and STEM currently requires excessive assumption and interpretation. Particularly given that gender representation is known to differ across fields often considered part of STEM, contributing to conflicting findings on gender underrepresentation (Cheryan et al. 2017), inconsistency in defining STEM has likely led to muddled interpretations of the literature at best. At worst, misleading implications about equity for women may have affected decisions to support their development and success in college. This brief aims to illuminate how differing STEM definitions may lead to varied results and potentially inconsistent conclusions, and to offer recommendations to the field for addressing this issue.

## Data and methods

We studied students from the NCES’ Education Longitudinal Study (ELS; 2002/12; Ingels et al. 2012). These data were collected via a multi-stage random sampling process, making the data nationally representative of 12th grade students in the USA. In other words, these findings generalize to all US high school seniors, examining specifically those who went on to enroll in college. We examined the relationship between gender and STEM bachelor’s degree attainment. STEM degree achievement was compared to earning a non-STEM degree or not attaining a degree through descriptive and regression-based analyses.

Multinomial logistic regression was used since the dependent degree variable had multiple categories (Long 1997). Running otherwise identical models, we compared five operational definitions of STEM majors. First, we defined STEM fields (a) based on the hard-soft paradigm distinction defined by Biglan (1973). Hard paradigm fields have a high degree of consensus about prevailing paradigms (e.g., physics or chemistry), while soft paradigm fields have a low degree of such consensus (e.g., anthropology or history). We then included (b) the operational definition provided by NCES that was adapted from the National Science and Mathematics Access to Retain Talent (SMART) Grant.^1^ Additionally, we used (c) NSF’s broad STEM definition which includes the social and behavioral sciences, as well as this NSF definition disaggregated into two mutually exclusive groups, including (d) science and engineering fields and (e) science and engineering-related fields as operationalized by NCES in the ELS dataset. We chose these definitions because they are either frequently used or, in the case of our STEM definition using Biglan types, often used to distinguish major types and disciplinary fields in higher education research. We acknowledge that these definitions are all US-based and other countries may have different typical examples of STEM definitions. Although this represents a limitation of this study, the main question of how differences in definition may alter conclusions of the research is valid in any context.

The five models comparing these STEM definitions included controls for gender, math self-efficacy, highest high school math course, math test score, race/ethnicity, socioeconomic status, delayed college entry, engaging in high impact college practices, and college GPA. Additional methodological details, including about the variables used, the codes from the Classification of Instructional Programs (CIP) that we used for our operationalization of STEM fields based on Biglan’s idea of hard paradigm, a description of how we handled missing data using multiple imputation, and information about the robustness checks we performed, can be found in the Additional file 1, along with full results for all five STEM definitions.

## Findings

**Fig. 1:**
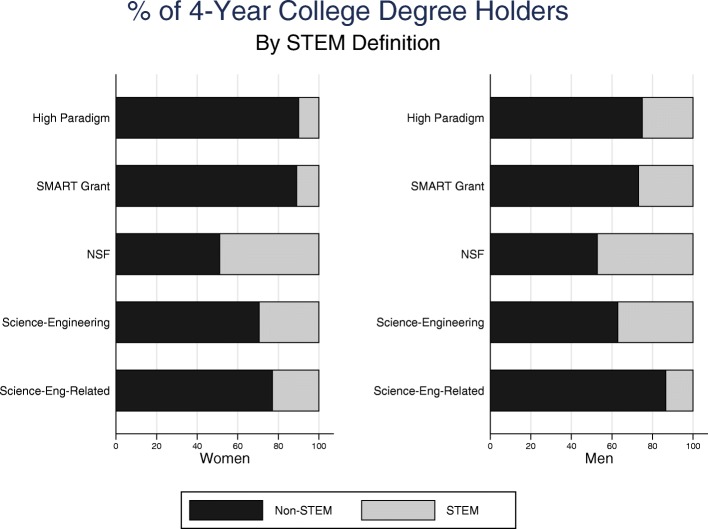
Percent of 4-year college degree holders, by gender, for each definition of STEM

Figure 1 illustrates varying gender representation in degree attainment, based on the different operationalizations of STEM. The broad NSF definition, which includes the social and behavioral sciences, showed the least gender difference. When broken down, however, the science and engineering portion of this definition showed slight underrepresentation of women. The science and engineering-*related* portion of the NSF definition was the only version resulting in a greater representation of women. The SMART Grant definition closely corresponded with Biglan’s concept of fields with high paradigm consensus, showing the largest underrepresentation of women in STEM.

As shown in Table 1, these gendered differences were sustained even controlling for other demographic, academic, and college-related factors. Again, the SMART definition was consistent with Biglan’s high paradigm concept; being a woman predicted similarly higher odds of non-STEM versus STEM undergraduate degree completion. However, NSF’s “science and engineering” operationalization had a smaller but statistically significant relationship, while using only the “science and engineering-related” fields revealed a negative relationship between being a woman and the odds of completing a non-STEM degree relative to a STEM degree. No gender difference was predicted when using the aggregated NSF definition with controls.

**Table 1:** Odds of attaining an undergraduate STEM degree—women vs. men—for five STEM definitions

STEM definition	No degree	Non-STEM	Odds of non-STEM compared to STEM degree
Biglan high paradigm	2.3**	2.7**	Larger positive
SMART Grant	1.9**	2.5**	Larger positive
NSF	0.8+	0.9	Statistically non-significant
Science and engineering	1.1	1.3*	Smaller positive
Science and engineering-related	0.6**	0.6**	Negative
Observations	7800	7800	

Source: Education Longitudinal Study (ELS; 2002/2012)

*Note*: Multinomial logistic regression results; odds ratios reported. All reported sample sizes are rounded to the nearest 10 in accordance with NCES restricted data license. Control variables used in all analyses are described in the Additional file 1

^**^p < 0.001; ^*^p < 0.01; ^+^*p* < 0.05

## Implications

The choice of a definition matters when drawing conclusions about gender and STEM degree completion. Our results suggest educators and researchers must be aware that sensitivity to STEM operationalization is necessary in order to apply results appropriately in practice and to test the validity of results. Variation across definitions is not inherently bad, but a lack of transparency about this facet of the research is likely to lead to confusion or error. While we investigated degree completion, educators and researchers need awareness that variation across STEM definitions has the potential to be different when other STEM-related outcomes are investigated. For example, whether researchers measure students’ first or last major in college may lead to different conclusions (Baum et al. 2015), in part due to students leaving STEM fields, including gender differences in attrition. These types of inequities continue beyond degree completion and with similar confusion based on STEM definition. Some claim that fewer women than men with STEM degrees actually work in STEM occupations after graduation (Beede et al. 2011), while others report the opposite—that women with STEM degrees work in STEM occupations more often—largely based on including health-related occupations in their definition of STEM (Funk and Parker 2018). A lack of clarity in the literature can lead to misunderstandings about the causes and consequences of STEM inequity (e.g., Riegle-Crumb and King 2010) as well as the experiences and engagement of STEM students. In turn, efforts aimed at addressing inequity in STEM have the potential to be based on faulty, or at least uncertain, foundations.

We found the size of the gender gap depends on the definition of STEM, consistent with existing research. At one university, for example, fewer women were found to be engineers, while women had equal representation in other STEM fields and in the whole university (using a STEM definition that did not include social sciences [Kokkelenberg and Sinha 2010]). Such results could be compared with a more inclusive STEM definition to determine the extent of this gap. Even rigorous studies finding gender underrepresentation would be strengthened further by checking STEM definition sensitivity (e.g., Riegle-Crumb and King 2010).

Given that only about a quarter of the studies in our content analysis used a common, externally referenced definition, and studies typically used only one definition, the extent to which results would be robust to other operational definitions remains unclear. This suggests caution is warranted when making comparisons across literature. The field should strive to find common categorizations of STEM that retain the legitimate variation in how STEM can and should be defined, while providing a basis for consistent comparison. For example, Baum et al. (2015) have suggested STEM-Core, STEM-SS (including the social sciences), and STEM-HealthTech (including the health professions and the science and engineering-related technologies). In a community college context, Lundy-Wagner and Chan (2016) have offered a STEM classification also distinguishing allied health and technology/technician fields. Further research is needed to confirm a set of definitions that would be most useful, and details of these definitions should be straightforwardly available to educators and researchers. Even more problematic than variation in operational definitions are those studies that did not clearly articulate how they defined STEM at all beyond the obvious and vague “science, technology, engineering and math,” which should be a basic expectation of STEM-related research. Without such clarity, efforts to address educational inequities in STEM may be guided by incorrect assumptions about the relevance of research results to particular support initiatives.

In summary, we recommend that STEM educators and researchers interpret findings with the understanding that what fields are considered STEM is inconsistent in the literature. We also recommend that practitioners and scholars researching STEM college students explicitly describe their STEM definition to enable comparability of findings and routinely analyze the sensitivity of results to alternate STEM definitions. Additionally, we recommend that the field articulate common STEM categorizations that retain legitimate variation while providing a basis for consistent comparison.

## Additional file

Additional file 1: Additional methodological details. (DOCX 75 kb)

## Abbreviations

ELS: Education Longitudinal Study
NCES: National Center for Education Statistics
NSF: National Science Foundation
SMART: Science and Mathematics Access to Retain Talent
STEM: Science, technology, engineering, and math

## Footnotes

1 For details about SMART Grants, see http://www2.ed.gov/programs/smart/
