# Supplementary material for: The influence of STEM definitions for research on women’s college attainment
Source: Int J STEM Educ. 2018 Nov 1;5(1):45. doi: 10.1186/s40594-018-0144-1 (PMC6310425; doi:10.1186/s40594-018-0144-1)
Supplement: Supplementary file 1 — Additional methodological details. (DOCX 75 kb) [file 40594_2018_144_MOESM1_ESM.docx]

**The Influence of STEM Definitions: Additional File**

Authors: Catherine A. Manly, University of Massachusetts Amherst

Ryan S. Wells, University of Massachusetts Amherst

Suzan Kommers, University of Massachusetts Amherst

Correspondence: cmanly@educ.umass.edu

Article: The Influence of STEM Definitions for Research on Women's College Attainment

Published: *International Journal of STEM Education*

This additional file includes information about the content analysis, the variables used in the regression analysis, our use of multiple imputation to handle missing data, the robustness checks we performed, a table showing the full results for all five models including the control variables, a full list of the articles included in the content analysis, and a list CIP codes included in our operationalization of STEM using Biglan’s (1973) hard paradigm concept.

**Table of Contents**

[Content Analysis Details 1](#_Toc523299794)

[Variables 2](#_Toc523299795)

[Multiple Imputation for Missing Data 2](#_Toc523299796)

[Robustness Checks 3](#_Toc523299797)

[Regression Analysis Results Including Controls 3](#_Toc523299798)

Table. [Odds of attaining an undergraduate STEM degree – women vs. men – for each operationalization of STEM, including all control variables (multinomial logistic regression results; odds ratios reported) 4](#_Toc523299800)

[References 6](#_Toc523299801)

Appendix A. [Articles Included in Content Analysis 7](#_Toc523299803)

Appendix B. [Hard paradigm CIP codes and corresponding occupation titles 11](#_Toc523299805)

# Content Analysis Details

The journal article content analysis, informed by the procedures for systematic literature reviews recommended by Moher, Liberati, Tetzlaff, and Altman (2009), included a search of the ERIC database on August 21 2018 using the following criteria:

- Search terms: STEM AND (gender OR women OR men)
- Full Text available in ERIC
- Peer Reviewed
- Date Published: January 2010 through July 2018
- Article Type: Journal Articles only
- Education Level: Higher Education, Postsecondary Education, Two Year Colleges
- Language: English

Result: 225 unduplicated articles identified through database screening

Additional search criteria imposed included:

- Journals ranked by the Scimago Journal Rank (SJR) ( <https://www.scimagojr.com/journalrank.php?category=3304> ) on August 14, 2018

Result: 111 articles are in ranked journals (114 excluded because in unranked journals)

- Quantitative empirical research
- Research using “STEM” in a multi-disciplinary way (i.e., single disciplinary studies excluded)
- Gender focus for research questions
- Postsecondary education focus for population studied

Result: 51 articles eligible for analysis (60 excluded as not meeting target content criteria)

See Appendix A for a complete list of the 51 articles analyzed.

# Variables

Our key independent variable was whether the student was a woman (F1SEX), and our control variables included: a measure of high school math self-efficacy developed by NCES (F1MATHSE); an indication of the highest level high school math course taken half a year or more (F1HIMATH); a measure of math ability through a standardized test administered by NCES during the 12^th^ grade year (F1TXMSTD); race and ethnicity as measured by whether the students were White, Asian, or of underrepresented racial/ethnic groups (F1RACE); family socioeconomic status (F1SES1); whether the student delayed entry to college (F3PSTIMING); engagement in what NCES defined as “high impact” practices during college (F3HIIMPACT; coded as 3 groups representing none, low, and high engagement levels); and their academic achievement measured by their overall college grade point average (F3TZGPAALL).

Our dependent variables included five operational definitions of STEM majors. First, we coded ELS’ degree fields a) based on the hard-soft paradigm distinction defined by Biglan (1973). See below for our operationalization. We then included b) a definition utilized by the SMART Grant (F3TZSTEM1CRED). Finally, we used c) NSF’s broad STEM definition (F3TZSTEM2CRED), as well as this definition disaggregated into d) science and engineering fields (F3TZSCENCRED), and e) science and engineering-related fields (F3TZSCENRCRED).^[[1]](#footnote-1)^

Our operationalization of STEM fields based on Biglan’s (1973) idea of hard paradigm includes the codes that are listed in Appendix B from the Classification of Instructional Programs (CIP) as hard paradigm fields.

# Multiple Imputation for Missing Data

As with any large-scale survey data collection, missing data were an issue. Most variables had under 20% missing, with up to 28% missing for the math self-efficacy variable. We chose to handle this missingness via the widely recommended multiple imputation method under the assumption that data were missing at random (Manly & Wells, 2015). Using the chained equations imputation method (via *mi impute chained* in Stata 14), and including the primary sampling unit, strata, and weights due to the complex design of the survey (Heeringa et al., 2010), we generated *m*=50 imputations, a number chosen because it was greater than the fraction of missing information for any analysis conducted. Imputed and observed values compared reasonably (van Buuren, 2012), and Rubin’s (1987) rules were used to pool results.

# Robustness Checks

We conducted several checks of the robustness of our results. Almost all listwise deletion (LD) results were similar to the results under multiple imputation (MI), and so multiply imputed results are presented given the expectation that they are less biased due to the presence of missing data. Only one result (that for no degree using the NSF definition) switched from being marginally significant under MI to not being significant under LD, but given our large sample size we consider these to be similar results. All effect sizes had less than a 0.2 difference.

We also checked whether the results differed if the imputed values on the dependent variable were dropped after multiple imputation was conducted, but found that this made little difference. One result (that for non-STEM degrees using the science-engineering definition) switched from being significant under MI to being only marginally significant when imputations for the dependent variable were dropped. All effect sizes had less than a 0.06 difference.

Additionally, we checked whether use of multiple or single imputation mattered for gender, by comparing use of NCES’ single imputation for gender to a multiply imputed gender variable. We found no substantial differences, and so we use NCES’ singly imputed gender variable.

We also evaluated results for any college attendees by combining 2- and 4-year institutions. The substantive conclusions one would draw were similar for both subsamples except for the NSF definition of STEM, where both odds ratios were significantly negative for females in the combined sample instead of not significant or only marginally so in just the 4-year sample. This result supports our assertion that the operationalization of STEM matters for drawing substantive conclusions regarding the relationship between gender and STEM degrees.

# Regression Analysis Results Including Controls

The following Table represents the full results of the analysis presented in Table 1 in the full paper.

## Table.

## Odds of attaining an undergraduate STEM degree – women vs. men – for each operationalization of STEM, including all control variables (multinomial logistic regression results; odds ratios reported)

|  | Biglan High Paradigm | | SMART Grant | | NSF | | Science & Engineering | | Science & Eng.-Related | |
| --- | --- | --- | --- | --- | --- | --- | --- | --- | --- | --- |
| Variable | No Degree | Non-STEM | No Degree | Non-STEM | No Degree | Non-STEM | No Degree | Non-STEM | No Degree | Non-STEM |
| Women | 2.341** | 2.720** | 1.946** | 2.526** | 0.803+ | 0.872 | 1.050 | 1.253* | 0.514** | 0.538** |
|  | (0.277) | (0.282) | (0.257) | (0.264) | (0.085) | (0.067) | (0.120) | (0.108) | (0.054) | (0.048) |
| Math self-efficacy | 0.873 | 0.788** | 0.890 | 0.785** | 1.021 | 0.891+ | 1.020 | 0.902+ | 1.018 | 0.949 |
|  | (0.063) | (0.051) | (0.067) | (0.048) | (0.059) | (0.040) | (0.067) | (0.041) | (0.063) | (0.046) |
| Highest high school math | 0.567** | 0.585** | 0.702* | 0.802 | 0.789* | 0.869 | 0.721* | 0.809+ | 0.840+ | 1.064 |
|  | (0.077) | (0.076) | (0.094) | (0.099) | (0.067) | (0.063) | (0.072) | (0.069) | (0.069) | (0.074) |
| Math test score | 0.688** | 0.625** | 0.559** | 0.543** | 0.807* | 0.779** | 0.662** | 0.613** | 1.198+ | 1.327** |
|  | (0.065) | (0.052) | (0.058) | (0.045) | (0.064) | (0.048) | (0.056) | (0.038) | (0.094) | (0.089) |
| Asian | 0.561* | 0.642* | 0.382** | 0.528** | 0.458** | 0.535** | 0.381** | 0.478** | 0.579** | 0.910 |
|  | (0.103) | (0.091) | (0.064) | (0.066) | (0.066) | (0.055) | (0.059) | (0.050) | (0.084) | (0.108) |
| Other race/ethnicity | 0.715+ | 0.755+ | 0.643* | 0.725+ | 0.775+ | 0.813+ | 0.629** | 0.681** | 1.021 | 1.150 |
|  | (0.102) | (0.098) | (0.103) | (0.099) | (0.092) | (0.077) | (0.083) | (0.072) | (0.121) | (0.112) |
| Socioeconomic status | 0.900 | 1.135 | 0.814+ | 1.125 | 0.764** | 1.064 | 0.690** | 0.909 | 0.884 | 1.323** |
|  | (0.081) | (0.085) | (0.075) | (0.081) | (0.060) | (0.059) | (0.059) | (0.055) | (0.070) | (0.082) |
| Delayed college entry | 2.609** | 1.008 | 5.222** | 1.668+ | 3.453** | 1.106 | 7.239** | 2.488** | 2.229** | 0.647* |
|  | (0.641) | (0.226) | (1.389) | (0.388) | (0.607) | (0.181) | (1.551) | (0.477) | (0.338) | (0.091) |
| High-impact college activities | 0.408** | 0.947 | 0.391** | 0.954 | 0.350** | 0.784** | 0.379** | 0.904 | 0.295** | 0.753** |
|  | (0.036) | (0.065) | (0.037) | (0.065) | (0.029) | (0.038) | (0.032) | (0.049) | (0.023) | (0.041) |
| College GPA | 0.308** | 0.885 | 0.165** | 0.967 | 0.174** | 0.941 | 0.141** | 0.859+ | 0.165** | 0.800* |
|  | (0.033) | (0.089) | (0.021) | (0.097) | (0.018) | (0.068) | (0.015) | (0.066) | (0.015) | (0.058) |
| Constant | 543.321** | 35.500** | 1,635.464** | 10.911** | 443.970** | 3.237** | 1,478.666** | 8.882** | 1,605.765** | 11.081** |
|  | (239.713) | (13.880) | (759.449) | (4.657) | (144.634) | (0.876) | (522.153) | (2.796) | (509.736) | (3.116) |
| Observations | 7,800 | | 7,800 | | 7,800 | | 7,800 | | 7,800 | |
| Log-likelihood [range] | -1,367,915 | | -1,243,900 | | -1,591,748 | | -1,460,240 | | -1,789,671 | |
|  | [-1,385,385,  -1,348,529] | | [-1,266,178,  -1,228,075] | | [-1,606,679,  -1,578,317] | | [-1,474,326, -  1,448,306] | | [-1,809,813,  -1,770,186] | |

Table (continued).

|  | Biglan High Paradigm | | SMART Grant | | | NSF | | | Science & Engineering | | | Science & Eng.-Related | | |  |
| --- | --- | --- | --- | --- | --- | --- | --- | --- | --- | --- | --- | --- | --- | --- | --- |
| Variable | No Degree | Non-STEM | No Degree | | Non-STEM | No Degree | | Non-STEM | No Degree | | Non-STEM | No Degree | | Non-STEM |  |
| McFadden Adj. R2 [range] | 0.191 | | | 0.273 | | | 0.206 | | | 0.241 | | | 0.275 | | |
|  | [0.182, 0.198] | | | [0.260, 0.281] | | | [0.199, 0.212] | | | [0.233, 0.247] | | | [0.268, 0.282] | | |
| F-adj. Mean Residual [range] | 35.408 | | | 28.345 | | | 37.915 | | | 46.749 | | | 45.384 | | |
|  | [21.822, 47.246] | | | [20.671, 38.442] | | | [30.415, 51.091] | | | [28.094, 61.378] | | | [35.603, 62.161] | | |
| F-adj. p-value [range] | 0.003 | | | 0.029 | | | 0.002 | | | <0.001 | | | <0.001 | | |
|  | [<0.001, 0.015] | | | [0.001, 0.192] | | | [<0.001, 0.016] | | | [<0.001, 0.031] | | | [<0.001, 0.003] | | |

Source: Education Longitudinal Study (ELS 2002/2012)

*Note*: All reported sample sizes are rounded to the nearest 10 in accordance with NCES restricted data license. The median and range of fit statistics are reported across the 50 imputations. **p<0.001; *p<0.01; +p<0.05.

# References

Biglan, A. (1973). The characteristics of subject matter in different academic areas. *Journal of Applied Psychology*, *57*(3), 195–203. doi:10.1037/h0034701

Heeringa, S., West, B. T., & Berglund, P. A. (2010). *Applied survey data analysis*. Boca Raton, FL: Chapman & Hall.

Manly, C. A., & Wells, R. S. (2015). Reporting the use of multiple imputation for missing data in higher education research. *Research in Higher Education*, *56*(4), 397–409. doi:10.1007/s11162-014-9344-9

Moher, D., Liberati, A., Tetzlaff, J., & Altman, D. G. (2009). Preferred reporting items for systematic reviews and meta-analyses: The PRISMA statement. *PLoS Medicine*, *6*(7). doi:10.1371/journal.pmed.1000097

Rubin, D. B. (1987). *Multiple imputation for nonresponse in surveys*. New York: Wiley.

van Buuren, S. (2012). *Flexible imputation of missing data*. Boca Raton, FL: CRC Press.

# Appendix A

# Articles Included in Content Analysis

Ackerman, P. L., Kanfer, R., & Beier, M. E. (2013). Trait complex, cognitive ability, and domain knowledge predictors of baccalaureate success, stem persistence, and gender differences. *Journal of Educational Psychology*, *105*, 911–927.

Aragón, O. R., Dovidio, J. F., & Graham, M. J. (2017). Colorblind and multicultural ideologies are associated with faculty adoption of inclusive teaching practices. *Journal of Diversity in Higher Education*, *10*(3), 201–215.

Baiduc, R. R., Drane, D., Beitel, G. J., & Flores, L. C. (2017). A research preparatory program for first-year college students: Student selection and preparation lead to persistence in research. *Innovative Higher Education*, *42*(3), 269–284.

Bieri Buschor, C., Berweger, S., Keck Frei, A., & Kappler, C. (2014). Majoring in stem--What accounts for women’s career decision making? A mixed methods study. *Journal of Educational Research*, *107*(3), 167–176.

Carnes, M., Devine, P. G., Isaac, C., Manwell, L. B., Ford, C. E., Byars-Winston, A., … Sheridan, J. (2012). Promoting institutional change through bias literacy. *Journal of Diversity in Higher Education*, *5*(2), 63–77.

Cerinsek, G., Hribar, T., Glodez, N., & Dolinsek, S. (2013). Which are my future career priorities and what influenced my choice of studying science, technology, engineering or mathematics? Some insights on educational choice--case of Slovenia. *International Journal of Science Education*, *35*, 2999–3025.

Chachashvili-Bolotin, S., Milner-Bolotin, M., & Lissitsa, S. (2016). Examination of factors predicting secondary students’ interest in tertiary STEM education. *International Journal of Science Education*, *38*(3), 366–390.

Chen, P. D., & Simpson, P. A. (2015). Does personality matter? Applying Holland’s typology to analyze students’ self-selection into science, technology, engineering, and mathematics majors. *Journal of Higher Education*, *86*(5), 725–750.

Dabney, K. P., & Tai, R. H. (2014). Comparative analysis of female physicists in the physical sciences: Motivation and background variables. *Physical Review Special Topics - Physics Education Research*, *10*(1), 1-10.

Dagley, M., Georgiopoulos, M., Reece, A., & Young, C. (2016). Increasing retention and graduation rates through a stem learning community. *Journal of College Student Retention: Research, Theory & Practice*, *18*(2), 167–182.

Dark, M. L. (2011). A photovoltaics module for incoming science, technology, engineering and mathematics undergraduates. *Physics Education*, *46*(3), 303–308.

Davies, P., Mangan, J., Hughes, A., & Slack, K. (2013). Labour market motivation and undergraduates’ choice of degree subject. *British Educational Research Journal*, *39*(2), 361–382.

Davison, M. L., Jew, G. B., & Davenport, E. C., Jr. (2014). Patterns of sat scores, choice of STEM major, and gender. *Measurement and Evaluation in Counseling and Development*, *47*(2), 118–126.

Espinosa, L. L. (2011). Pipelines and pathways: Women of color in undergraduate STEM majors and the college experiences that contribute to persistence. *Harvard Educational Review*, *81*(2), 209–241.

Gayles, J. G., & Ampaw, F. (2014). The impact of college experiences on degree completion in STEM fields at four-year institutions: Does gender matter? *Journal of Higher Education*, *85*(4), 439–468.

Heilbronner, N. N. (2011). Stepping onto the STEM pathway: Factors affecting talented students’ declaration of STEM majors in college. *Journal for the Education of the Gifted*, *34*(6), 876–899.

Heilbronner, N. N. (2013). The STEM pathway for women: What has changed? *Gifted Child Quarterly*, *57*(1), 39–55.

Hernandez, P. R., Schultz, P. W., Estrada, M., Woodcock, A., & Chance, R. C. (2013). Sustaining optimal motivation: A longitudinal analysis of interventions to broaden participation of underrepresented students in STEM. *Journal of Educational Psychology*, *105*, 89–107.

Hübner, N., Wille, E., Cambria, J., Oschatz, K., Nagengast, B., & Trautwein, U. (2017). Maximizing gender equality by minimizing course choice options? Effects of obligatory coursework in math on gender differences in STEM. *Journal of Educational Psychology*, *109*, 993–1009.

Johnson, D. R. (2012). Campus racial climate perceptions and overall sense of belonging among racially diverse women in STEM majors. *Journal of College Student Development*, *53*(2), 336–346.

Katz, L. A., Aloisio, K. M., Horton, N. J., Ly, M., Pruss, S., Queeney, K., … DiBartolo, P. M. (2017). A program aimed toward inclusive excellence for underrepresented undergraduate women in the sciences. *CBE - Life Sciences Education*, *16*(1), 1-9.

Kerr, B. A., Multon, K. D., Syme, M. L., Fry, N. M., Owens, R., Hammond, M., & Robinson-Kurpius, S. (2012). Development of the distance from privilege measures: A tool for understanding the persistence of talented women in STEM. *Journal of Psychoeducational Assessment*, *30*(1), 88–102.

Kimmel, L. G., Miller, J. D., & Eccles, J. S. (2012). Do the paths to STEMM professions differ by gender? *Peabody Journal of Education*, *87*(1), 92–113.

Kordaki, M., & Berdousis, I. (2017). Computing and STEM in Greece: Gender representation of students and teachers during the decade 2002/2012. *Education and Information Technologies*, *22*(1), 101–124.

Korpershoek, H., Kuyper, H., Bosker, R., & van der Werf, G. (2013). Students’ preconceptions and perceptions of science-oriented studies. *International Journal of Science Education*, *35*, 2356–2375.

Lin, K.-Y., & Williams, P. J. (2016). Taiwanese preservice teachers’ science, technology, engineering, and mathematics teaching intention. *International Journal of Science and Mathematics Education*, *14*(6), 1021–1036.

Nosek, B. A., & Smyth, F. L. (2011). Implicit social cognitions predict sex differences in math engagement and achievement. *American Educational Research Journal*, *48*, 1125–1156.

Novak, H., Paguyo, C., & Siller, T. (2016). Examining the impact of the engineering successful/unsuccessful grading (SUG) program on student retention. *Journal of College Student Retention: Research, Theory & Practice*, *18*(1), 83–108.

Olitsky, N. H. (2014). How do academic achievement and gender affect the earnings of STEM majors? A propensity score matching approach. *Research in Higher Education*, *55*(3), 245–271.

Park, G., Lubinski, D., & Benbow, C. P. (2013). When less is more: Effects of grade skipping on adult stem productivity among mathematically precocious adolescents. *Journal of Educational Psychology*, *105*, 176–198.

Pasha-Zaidi, N., & Afari, E. (2016). Gender in STEM education: An exploratory study of student perceptions of math and science instructors in the United Arab Emirates. *International Journal of Science and Mathematics Education*, *14*(7), 1215–1231.

Rabitoy, E. R., Hoffman, J. L., & Person, D. R. (2015). Supplemental instruction: The effect of demographic and academic preparation variables on community college student academic achievement in STEM-related fields. *Journal of Hispanic Higher Education*, *14*(3), 240–255.

Riegle-Crumb, C., & King, B. (2010). Questioning a white male advantage in STEM: Examining disparities in college major by gender and race/ethnicity. *Educational Researcher*, *39*(9), 656–664.

Riegle-Crumb, C., King, B., Grodsky, E., & Muller, C. (2012). The more things change, the more they stay the same? Prior achievement fails to explain gender inequality in entry into STEM college majors over time. *American Educational Research Journal*, *49*, 1048–1073.

Rincón, B. E., & Lane, T. B. (2017). Latin@s in science, technology, engineering, and mathematics (STEM) at the intersections. *Equity & Excellence in Education*, *50*(2), 182–195.

Sadler, P. M., Sonnert, G., Hazari, Z., & Tai, R. (2012). Stability and volatility of STEM career interest in high school: A gender study. *Science Education*, *96*(3), 411–427.

Sahin, A., Ekmekci, A., & Waxman, H. C. (2017). The relationships among high school STEM learning experiences, expectations, and mathematics and science efficacy and the likelihood of majoring in STEM in college. *International Journal of Science Education*, *39*(11), 1549–1572.

Sahin, A., Gulacar, O., & Stuessy, C. (2015). High school students’ perceptions of the effects of international science Olympiad on their STEM career aspirations and twenty-first century skill development. *Research in Science Education*, *45*(6), 785–805.

Sax, L. J., Kanny, M. A., Riggers-Piehl, T. A., Whang, H., & Paulson, L. N. (2015). “But I’m not good at math”: The changing salience of mathematical self-concept in shaping women’s and men’s STEM aspirations. *Research in Higher Education*, *56*(8), 813–842.

Simon, R. A., Aulls, M. W., Dedic, H., Hubbard, K., & Hall, N. C. (2015). Exploring student persistence in STEM programs: A motivational model. *Canadian Journal of Education*, *38*(1), 1-27.

Simon, R. M., Wagner, A., & Killion, B. (2017). Gender and choosing a STEM major in college: Femininity, masculinity, chilly climate, and occupational values. *Journal of Research in Science Teaching*, *54*(3), 299–323.

Smith, E. (2011). Women into science and engineering? Gendered participation in higher education STEM subjects. *British Educational Research Journal*, *37*(6), 993–1014.

Solberg, V. S., Kimmel, L. G., & Miller, J. D. (2012). Pathways to STEMM support occupations. *Peabody Journal of Education*, *87*(1), 77–91.

Szelényi, K., Denson, N., & Inkelas, K. K. (2013). Women in STEM majors and professional outcome expectations: The role of living-learning programs and other college environments. *Research in Higher Education*, *54*(8), 851–873.

Szelenyi, K., & Inkelas, K. K. (2011). The role of living-learning programs in women’s plans to attend graduate school in STEM fields. *Research in Higher Education*, *52*(4), 349–369.

Toven-Lindsey, B., Levis-Fitzgerald, M., Barber, P. H., & Hasson, T. (2015). Increasing persistence in undergraduate science majors: A model for institutional support of underrepresented students. *CBE - Life Sciences Education*, *14*(2), 1-12.

Wang, X. (2013). Why students choose STEM majors: Motivation, high school learning, and postsecondary context of support. *American Educational Research Journal*, *50*, 1081–1121.

Wladis, C., Conway, K. M., & Hachey, A. C. (2015). The online STEM classroom--Who succeeds? An exploration of the impact of ethnicity, gender, and non-traditional student characteristics in the community college context. *Community College Review*, *43*(2), 142–164.

Wladis, C., Hachey, A. C., & Conway, K. M. (2015). The representation of minority, female, and non-traditional stem majors in the online environment at community colleges: A nationally representative study. *Community College Review*, *43*(1), 89–114.

Xu, Y. (2015). Focusing on women in STEM: A longitudinal examination of gender-based earning gap of college graduates. *Journal of Higher Education*, *86*(4), 489–523.

Zhang, L. (2011). Does merit-based aid affect degree production in STEM fields? Evidence from Georgia and Florida. *Journal of Higher Education*, *82*(4), 389–415.

# Appendix B

# Hard paradigm CIP codes and corresponding occupation titles

| Hard Paradigm CIP Code | CIP Occupation Title |
| --- | --- |
| 01.0308 | Agroecology and Sustainable Agriculture |
| 01.0508 | Taxidermy/Taxidermist |
| 01.0603 | Ornamental Horticulture |
| 01.0901 | Animal Sciences, General |
| 01.0903 | Animal Health |
| 01.0904 | Animal Nutrition |
| 01.0905 | Dairy Science |
| 01.0907 | Poultry Science |
| 01.0999 | Animal Sciences, Other |
| 01.1001 | Food Science |
| 01.1099 | Food Science and Technology, Other. |
| 01.1101 | Plant Sciences, General |
| 01.1102 | Agronomy & Crop Science |
| 01.1103 | Horticultural Science |
| 01.1106 | Range Science & Management |
| 01.1199 | Plant Sciences, Other |
| 01.1201 | Soil Science & Agronomy, General |
| 01.1202 | Soil Chemistry & Physics |
| 01.1203 | Soil Microbiology |
| 01.9999 | Agriculture, Agricultural Operations, & Related Sciences, Other. |
| 03.0103 | Environmental Studies |
| 03.0104 | Environmental Science |
| 03.0502 | Forest Sciences |
| 03.0509 | Wood Science & Wood Products/Pulp & Paper Technology |
| 03.0601 | Wildlife & Wildlands Science & Management |
| 03.9999 | Natural Resources & Conservation, Other |
| 11.0101 | Computer & Information Sciences, General |
| 11.0102 | Artificial Intelligence & Robotics |
| 11.0103 | Information Technology |
| 11.0199 | Computer and Information Sciences, Other. |
| 11.0201 | Computer Programming/Programmer, General |
| 11.0202 | Computer Programming, Specific Applications |
| 11.0203 | Computer Programming, Vendor/Product Certification |
| 11.0301 | Data Processing & Data Processing Technology/Technician |
| 11.0401 | Information Science/Studies |
| 11.0501 | Computer Systems Analysis/Analyst |
| 11.0601 | Data Entry/Microcomputer Applications |
| 11.0701 | Computer Science |
| 11.0802 | Data Modeling/Warehousing and Database Administration |
| 11.0899 | Computer Software and Media Applications, Other. |
| 11.0901 | Computer Systems Networking & Telecommunications |
| 11.1001 | System Administration/Administrator |
| 11.1002 | System, Networking, & LAN/WAN Management/Manager |
| 11.1003 | Computer & Information Systems Security |
| 11.1099 | Computer/Information Technology Services Administration and Management, Other. |
| 11.9999 | Computer & Information Sciences & Support Services, Other |
| 14.0101 | Engineering, General |
| 14.0201 | Aerospace, Aeronautical & Astronautical Engineering |
| 14.0301 | Agricultural/Biological Engineering & Bioengineering |
| 14.0401 | Architectural Engineering |
| 14.0501 | Biomedical/Medical Engineering |
| 14.0601 | Ceramic Sciences & Engineering |
| 14.0701 | Chemical Engineering |
| 14.0702 | Chemical and Biomolecular Engineering. |
| 14.0801 | Civil Engineering, General |
| 14.0802 | Geotechnical Engineering |
| 14.0803 | Structural Engineering |
| 14.0804 | Transportation & Highway Engineering |
| 14.0805 | Water Resources Engineering |
| 14.0899 | Civil Engineering, Other |
| 14.0901 | Computer Engineering, General |
| 14.0902 | Computer Hardware Engineering |
| 14.0903 | Computer Software Engineering |
| 14.0999 | Computer Engineering, Other |
| 14.1001 | Electrical, Electronics & Communications Engineering |
| 14.1099 | Electrical, Electronics and Communications Engineering, Other. |
| 14.1101 | Engineering Mechanics |
| 14.1201 | Engineering Physics |
| 14.1301 | Engineering Science |
| 14.1401 | Environmental/Environmental Health Engineering |
| 14.1801 | Materials Engineering |
| 14.1901 | Mechanical Engineering |
| 14.2001 | Metallurgical Engineering |
| 14.2101 | Mining & Mineral Engineering |
| 14.2201 | Naval Architecture & Marine Engineering |
| 14.2301 | Nuclear Engineering |
| 14.2401 | Ocean Engineering |
| 14.2501 | Petroleum Engineering |
| 14.2701 | Systems Engineering |
| 14.2801 | Textile Sciences & Engineering |
| 14.3101 | Materials Science |
| 14.3201 | Polymer/Plastics Engineering |
| 14.3301 | Construction Engineering |
| 14.3401 | Forest Engineering |
| 14.3501 | Industrial Engineering |
| 14.3601 | Manufacturing Engineering |
| 14.3701 | Operations Research |
| 14.3801 | Surveying Engineering |
| 14.3901 | Geological/Geophysical Engineering |
| 14.4301 | Biochemical Engineering. |
| 14.4501 | Biological/Biosystems Engineering. |
| 14.9999 | Engineering, Other |
| 15.0000 | Engineering Technology, General. |
| 15.0101 | Architectural Engineering Technology/Technician |
| 15.0201 | Civil Engineering Technology/Technician |
| 15.0303 | Electrical, Electronic & Communications Engineering Technology/Technician |
| 15.0304 | Laser & Optical Technology/Technician |
| 15.0305 | Telecommunications Technology/Technician |
| 15.0399 | Electrical & Electronic Engineering Technologies/Technicians, Other |
| 15.0401 | Biomedical Technology/Technician |
| 15.0403 | Electromechanical Technology/Electromechanical Engineering Technology |
| 15.0404 | Instrumentation Technology/Technician |
| 15.0405 | Robotics Technology/Technician |
| 15.0406 | Automation Engineer Technology/Technician. |
| 15.0499 | Electromechanical & Instrumentation & Maintenance Technologies/Technicians, Other |
| 15.0501 | Heating, Air Conditioning & Refrigeration Technology/Technician (ACH/ACR/ACHR/HRAC/HVAC/AC Technol |
| 15.0503 | Energy Management & Systems Technology/Technician |
| 15.0505 | Solar Energy Technology/Technician |
| 15.0506 | Water Quality & Wastewater Treatment Management & Recycling Technology/Technician |
| 15.0507 | Environmental Engineering Technology/Environmental Technology |
| 15.0508 | Hazardous Materials Management & Waste Technology/Technician |
| 15.0599 | Environmental Control Technologies/Technicians, Other |
| 15.0607 | Plastics Engineering Technology/Technician |
| 15.0611 | Metallurgical Technology/Technician |
| 15.0704 | Hazardous Materials Information Systems Technology/Technician |
| 15.0801 | Aeronautical/Aerospace Engineering Technology/Technician |
| 15.0803 | Automotive Engineering Technology/Technician |
| 15.0805 | Mechanical Engineering/Mechanical Technology/Technician |
| 15.0899 | Mechanical Engineering Related Technology/Technician, Other |
| 15.0901 | Mining Technology/Technician |
| 15.0903 | Petroleum Technology/Technician |
| 15.0999 | Mining & Petroleum Technologies/Technicians, Other |
| 15.1401 | Nuclear Engineering Technology/Technician |
| 15.9999 | Engineering Related Technologies/Technicians, Other |
| 19.0504 | Human Nutrition |
| 26.0101 | Biology/Biological Sciences, General |
| 26.0102 | Biomedical Sciences, General |
| 26.0202 | Biochemistry |
| 26.0203 | Biophysics |
| 26.0204 | Molecular Biology |
| 26.0205 | Molecular Biochemistry |
| 26.0206 | Molecular Biophysics |
| 26.0207 | Structural Biology |
| 26.0208 | Photobiology |
| 26.0209 | Radiation Biology/Radiobiology |
| 26.0210 | Biochemistry/Biophysics & Molecular Biology |
| 26.0301 | Botany/Plant Biology |
| 26.0305 | Plant Pathology/Phytopathology |
| 26.0307 | Plant Physiology |
| 26.0308 | Plant Molecular Biology |
| 26.0399 | Botany/Plant Biology, Other |
| 26.0401 | Cell/Cellular Biology & Histology |
| 26.0403 | Anatomy |
| 26.0404 | Developmental Biology & Embryology |
| 26.0405 | Neuroanatomy |
| 26.0406 | Cell/Cellular & Molecular Biology |
| 26.0407 | Cell Biology & Anatomy |
| 26.0499 | Cell/Cellular Biology & Anatomical Sciences, Other |
| 26.0502 | Microbiology, General |
| 26.0503 | Medical Microbiology & Bacteriology |
| 26.0504 | Virology |
| 26.0505 | Parasitology |
| 26.0506 | Mycology |
| 26.0507 | Immunology |
| 26.0508 | Microbiology and Immunology. |
| 26.0599 | Microbiological Sciences and Immunology, Other. |
| 26.0701 | Zoology/Animal Biology |
| 26.0702 | Entomology |
| 26.0707 | Animal Physiology |
| 26.0708 | Animal Behavior & Ethology |
| 26.0709 | Wildlife Biology |
| 26.0799 | Zoology/Animal Biology, Other |
| 26.0801 | Genetics, General |
| 26.0802 | Molecular Genetics |
| 26.0803 | Microbial & Eukaryotic Genetics |
| 26.0804 | Animal Genetics |
| 26.0805 | Plant Genetics |
| 26.0806 | Human/Medical Genetics |
| 26.0807 | Genome Sciences/Genomics. |
| 26.0899 | Genetics, Other |
| 26.0901 | Physiology, General |
| 26.0902 | Molecular Physiology |
| 26.0903 | Cell Physiology |
| 26.0904 | Endocrinology |
| 26.0905 | Reproductive Biology |
| 26.0906 | Neurobiology & Neurophysiology |
| 26.0907 | Cardiovascular Science |
| 26.0908 | Exercise Physiology |
| 26.0909 | Vision Science/Physiological Optics |
| 26.0910 | Pathology/Experimental Pathology |
| 26.0911 | Oncology & Cancer Biology |
| 26.0999 | Physiology, Pathology, & Related Sciences, Other |
| 26.1001 | Pharmacology |
| 26.1002 | Molecular Pharmacology |
| 26.1003 | Neuropharmacology |
| 26.1004 | Toxicology |
| 26.1005 | Molecular Toxicology |
| 26.1006 | Environmental Toxicology |
| 26.1007 | Pharmacology & Toxicology |
| 26.1099 | Pharmacology & Toxicology, Other |
| 26.1101 | Biometry/Biometrics |
| 26.1102 | Biostatistics |
| 26.1103 | Bioinformatics |
| 26.1104 | Computational Biology. |
| 26.1199 | Biomathematics and Bioinformatics, Other |
| 26.1201 | Biotechnology |
| 26.1301 | Ecology |
| 26.1302 | Marine Biology & Biological Oceanography |
| 26.1303 | Evolutionary Biology |
| 26.1304 | Aquatic Biology/Limnology |
| 26.1305 | Environmental Biology |
| 26.1306 | Population Biology |
| 26.1307 | Conservation Biology |
| 26.1308 | Systematic Biology/Biological Systematics |
| 26.1309 | Epidemiology |
| 26.1310 | Ecology and Evolutionary Biology. |
| 26.1399 | Ecology, Evolution, & Systematics, Other |
| 26.1501 | Neuroscience. |
| 26.1503 | Neurobiology and Anatomy. |
| 26.9999 | Biological & Biomedical Sciences, Other |
| 27.0101 | Mathematics, General |
| 27.0102 | Algebra & Number Theory |
| 27.0103 | Analysis & Functional Analysis |
| 27.0104 | Geometry/Geometric Analysis |
| 27.0105 | Topology & Foundations |
| 27.0199 | Mathematics, Other |
| 27.0301 | Applied Mathematics |
| 27.0303 | Computational Mathematics |
| 27.0399 | Applied Mathematics, Other |
| 27.0501 | Statistics, General |
| 27.0502 | Mathematical Statistics & Probability |
| 27.0503 | Mathematics and Statistics. |
| 27.0599 | Statistics, Other |
| 27.9999 | Mathematics & Statistics, Other |
| 29.0207 | Cyber/Electronic Operations and Warfare. |
| 29.0301 | Combat Systems Engineering. |
| 29.0399 | Military Applied Sciences, Other |
| 29.0402 | Air and Space Operations Technology. |
| 29.0404 | Explosive Ordinance/Bomb Disposal. |
| 29.0406 | Military Information Systems Technology. |
| 30.0000 | Multi-/Interdisciplinary Studies, General. |
| 30.0101 | Biological & Physical Sciences |
| 30.0601 | Systems Science and Theory |
| 30.0801 | Mathematics & Computer Science |
| 30.1001 | Biopsychology |
| 30.1501 | Science, Technology & Society |
| 30.1601 | Accounting & Computer Science |
| 30.1801 | Natural Sciences |
| 30.1901 | Nutrition Sciences |
| 30.2401 | Neuroscience |
| 30.2501 | Cognitive Science |
| 30.2701 | Human Biology. |
| 30.3001 | Computational Science |
| 30.3101 | Human Computer Interaction. |
| 30.3201 | Marine Sciences |
| 30.3301 | Sustainability Studies |
| 30.9999 | Multi-/Interdisciplinary Studies, Other. |
| 40.0101 | Physical Sciences |
| 40.0201 | Astronomy |
| 40.0202 | Astrophysics |
| 40.0203 | Planetary Astronomy & Science |
| 40.0299 | Astronomy and Astrophysics, Other |
| 40.0401 | Atmospheric Sciences & Meteorology, General |
| 40.0402 | Atmospheric Chemistry & Climatology |
| 40.0403 | Atmospheric Physics & Dynamics |
| 40.0404 | Meteorology |
| 40.0499 | Atmospheric Sciences & Meteorology, Other |
| 40.0501 | Chemistry, General |
| 40.0502 | Analytical Chemistry |
| 40.0503 | Inorganic Chemistry |
| 40.0504 | Organic Chemistry |
| 40.0506 | Physical & Theoretical Chemistry |
| 40.0507 | Polymer Chemistry |
| 40.0508 | Chemical Physics |
| 40.0599 | Chemistry, Other |
| 40.0601 | Geology/Earth Science, General |
| 40.0602 | Geochemistry |
| 40.0603 | Geophysics & Seismology |
| 40.0604 | Paleontology |
| 40.0605 | Hydrology & Water Resources Science |
| 40.0606 | Geochemistry & Petrology |
| 40.0607 | Oceanography, Chemical & Physical |
| 40.0699 | Geological & Earth Sciences/Geosciences, Other |
| 40.0801 | Physics, General |
| 40.0802 | Atomic/Molecular Physics |
| 40.0804 | Elementary Particle Physics |
| 40.0805 | Plasma & High-Temperature Physics |
| 40.0806 | Nuclear Physics |
| 40.0807 | Optics/Optical Sciences |
| 40.0808 | Solid State & Low-Temperature Physics |
| 40.0809 | Acoustics |
| 40.0810 | Theoretical & Mathematical Physics |
| 40.0899 | Physics, Other |
| 40.9999 | Physical Sciences, Other |
| 41.0000 | Science Technologies/Technicians, General. |
| 41.0101 | Biology Technician/Biotechnology Laboratory Technician |
| 41.0204 | Industrial Radiologic Technology/Technician |
| 41.0205 | Nuclear/Nuclear Power Technology/Technician |
| 41.0299 | Nuclear & Industrial Radiologic Technologies/Technicians, Other |
| 41.0301 | Chemical Technology/Technician |
| 41.0399 | Physical Science Technologies/Technicians, Other |
| 41.9999 | Science Technologies/Technicians, Other |
| 43.0106 | Forensic Science & Technology |
| 43.0203 | Fire Science/Firefighting |
| 43.0204 | Fire Systems Technology. |
| 50.0404 | Industrial Design |
| 51.0201 | Communication Disorders, General |
| 51.0202 | Audiology/Audiologist & Hearing Sciences |
| 51.0203 | Speech-Language Pathology/Pathologist |
| 51.0204 | Audiology/Audiologist & Speech-Language Pathology/Pathologist |
| 51.0299 | Communication Disorders Sciences & Services, Other |
| 51.0401 | Dentistry (DDS, DMD) |
| 51.0501 | Dental Clinical Sciences, General (MS, PhD) |
| 51.0502 | Advanced General Dentistry (Cert, MS, PhD) |
| 51.0503 | Oral Biology & Pathology (MS, PhD) |
| 51.0506 | Endodontics/Endodontology (Cert, MS, PhD) |
| 51.0507 | Oral/Maxillofacial Surgery (Cert, MS, PhD) |
| 51.0508 | Orthodontics/Orthodontology (Cert, MS, PhD) |
| 51.0509 | Pediatric Dentistry/Pedodontics (Cert, MS, PhD) |
| 51.0510 | Periodontics/Periodontology (Cert, MS, PhD) |
| 51.0511 | Prosthodontics/Prosthodontology (Cert, MS, PhD) |
| 51.0599 | Advanced/Graduate Dentistry & Oral Sciences, Other |
| 51.1201 | Medicine (MD) |
| 51.1401 | Medical Scientist (MS, PhD) |
| 51.2001 | Pharmacy (PharmD, BS/BPharm) |
| 51.2003 | Pharmaceutics & Drug Design (MS, PhD) |
| 51.2004 | Medicinal & Pharmaceutical Chemistry (MS, PhD) |
| 51.2005 | Natural Products Chemistry & Pharmacognosy (MS, PhD) |
| 51.2099 | Pharmacy, Pharmaceutical Sciences, & Administration, Other |
| 51.2205 | Health/Medical Physics |
| 51.2401 | Veterinary Medicine (DVM) |
| 51.2501 | Veterinary Sciences/Veterinary Clinical Sciences, General (Cert, MS, PhD) |
| 51.2502 | Veterinary Anatomy (Cert, MS, PhD) |
| 51.2503 | Veterinary Physiology (Cert, MS, PhD) |
| 51.2504 | Veterinary Microbiology & Immunobiology (Cert, MS, PhD) |
| 51.2505 | Veterinary Pathology & Pathobiology (Cert, MS, PhD) |
| 51.2506 | Veterinary Toxicology & Pharmacology (Cert, MS, PhD) |
| 51.2507 | Large Animal/Food Animal & Equine Surgery & Medicine (Cert, MS, PhD) |
| 51.2508 | Small/Companion Animal Surgery & Medicine (Cert, MS, PhD) |
| 51.2509 | Comparative & Laboratory Animal Medicine (Cert, MS, PhD) |
| 51.2510 | Veterinary Preventive Medicine Epidemiology, & Public Health (Cert, MS, PhD) |
| 51.2511 | Veterinary Infectious Diseases (Cert, MS, PhD) |
| 51.2599 | Veterinary Biomedical & Clinical Sciences, Other (Cert, MS. PhD) |
| 52.1304 | Actuarial Science |
| 60.0101 | Dental/Oral Surgery Specialty |
| 60.0201 | Aerospace Medicine |
| 60.0203 | Anesthesiology |
| 60.0204 | Blood Banking |
| 60.0205 | Cardiology |
| 60.0206 | Chemical Pathology |
| 60.0207 | Child/Pediatric Neurology |
| 60.0209 | Colon & Rectal Surgery |
| 60.0210 | Critical Care Anesthesiology |
| 60.0211 | Critical Care Medicine |
| 60.0212 | Critical Care Surgery |
| 60.0213 | Dermatology |
| 60.0214 | Dermatopathology |
| 60.0215 | Diagnostic Radiology |
| 60.0216 | Emergency Medicine |
| 60.0217 | Endocrinology & Metabolism |
| 60.0218 | Family Medicine |
| 60.0219 | Forensic Pathology |
| 60.0220 | Gastroenterology |
| 60.0221 | General Surgery |
| 60.0222 | Geriatric Medicine |
| 60.0223 | Hand Surgery |
| 60.0224 | Hematology |
| 60.0225 | Hematological Pathology |
| 60.0226 | Immunopathology |
| 60.0227 | Infectious Disease |
| 60.0228 | Internal Medicine |
| 60.0229 | Laboratory Medicine |
| 60.0230 | Musculoskeletal Oncology |
| 60.0231 | Neonatal-Perinatal Medicine |
| 60.0232 | Nephrology |
| 60.0233 | Neurological Surgery/Neurosurgery |
| 60.0234 | Neurology |
| 60.0235 | Neuropathology |
| 60.0236 | Nuclear Medicine |
| 60.0237 | Nuclear Radiology |
| 60.0238 | Obstetrics & Gynecology |
| 60.0239 | Occupational Medicine |
| 60.0240 | Oncology |
| 60.0241 | Ophthalmology |
| 60.0242 | Orthopedics/Orthopedic Surgery |
| 60.0243 | Otolaryngology |
| 60.0244 | Pathology |
| 60.0245 | Pediatric Cardiology |
| 60.0246 | Pediatric Endocrinology |
| 60.0247 | Pediatric Hemato-Oncology |
| 60.0248 | Pediatric Nephrology |
| 60.0249 | Pediatric Orthopedics |
| 60.0250 | Pediatric Surgery |
| 60.0251 | Pediatrics |
| 60.0252 | Physical & Rehabilitation Medicine |
| 60.0253 | Plastic Surgery |
| 60.0254 | Preventive Medicine |
| 60.0257 | Pulmonary Disease |
| 60.0258 | Radiation Oncology |
| 60.0259 | Radioisotopic Pathology |
| 60.0260 | Rheumatology |
| 60.0262 | Thoracic Surgery |
| 60.0263 | Urology |
| 60.0264 | Vascular Surgery |
| 60.0265 | Adult Reconstructive Orthopedics (Orthopedic Surgery) |
| 60.0266 | Child Neurology |
| 60.0267 | Cytopathology |
| 60.0268 | Geriatric Medicine (Internal Medicine) |
| 60.0269 | Pediatric Urology |
| 60.0271 | Orthopedic Surgery of the Spine |
| 60.0301 | Veterinary Anesthesiology |
| 60.0302 | Veterinary Dentistry |
| 60.0303 | Veterinary Dermatology |
| 60.0305 | Veterinary Internal Medicine |
| 60.0306 | Laboratory Animal Medicine |
| 60.0307 | Veterinary Microbiology |
| 60.0309 | Veterinary Ophthalmology |
| 60.0310 | Veterinary Pathology |
| 60.0312 | Veterinary Preventive Medicine |
| 60.0313 | Veterinary Radiology |
| 60.0314 | Veterinary Surgery |
| 60.0316 | Veterinary Toxicology |
| 60.0317 | Zoological Medicine |

1. For details about these variables, see https://nces.ed.gov/datalab/powerstats/pdf/els2002sophomores_subject.pdf [↑](#footnote-ref-1)
